# *Codonopsis pilosula* inulin-type fructan CPA ameliorates diarrhea-predominant irritable bowel syndrome via regulation of ER stress-induced autophagy and modulation of gut microbiota

**DOI:** 10.3389/fphar.2026.1715972

**Published:** 2026-09-22

**Authors:** Linglin Tian, Yunuan Bai, Shiqi Wang, Changjian Wang, Jianping Gao, Jiangtao Zhou, Wenping Xiang

**Affiliations:** 1 Department of Gastroenterology, First Hospital of Shanxi Medical University, Taiyuan, China; 2 School of Pharmacy, Shanxi Medical University, Taiyuan, China; 3 Medicinal Basic Research Innovation Center of Chronic Kidney Disease, Ministry of Education, Shanxi Medical University, Taiyuan, China; 4 Baotou Mongolian and Traditional Chinese Medicine Hospital, Baotou, China

**Keywords:** autophagy, ER stress, gut microbiota, IBS-D, inulin-type fructan CP-A, WGCNA

## Abstract

Diarrhea-predominant irritable bowel syndrome (IBS-D) is characterized by diarrhea, abdominal pain, and recurrent symptoms, posing significant clinical challenges. This study utilized the GSE36701 dataset from the Gene Expression Omnibus (GEO) and applied weighted gene co-expression network analysis (WGCNA) to identify IBS-D-associated genes, with a specific focus on endoplasmic reticulum (ER) stress. *Codonopsis pilosula*, a traditional Chinese medicinal herb, and its active inulin-type fructan monomer CPA, exhibit potential gastrointestinal protective effects. This study evaluated the therapeutic effects of CPA both *in vivo*, using an IBS-D model induced by senna leaf combined with chronic unpredictable stimuli, and *in vitro*, using a lipopolysaccharide (LPS)-induced IBS-D model in NCM460 cells. Hematoxylin-Eosin Staining (H&E), Alcian Blue-Periodic Acid-Schiff Staining (AB-PAS), immunohistochemistry (IHC), Immunofluorescence (IF), Elisa, qRT-PCR and Western blots were used to detect relative indices in this research. Our results indicated that CPA treatment improved IBS-D symptoms, including increased body weight, reduced abdominal withdrawal reflex, and normalized stool consistency. Histological analysis revealed improved colonic crypt and gland morphology, reduced inflammatory cell infiltration, and increased goblet cell numbers. CPA also upregulated tight junction protein expression, anti-inflammatory cytokines, and neuroeffector neuropeptide Y (NP-Y) levels. In addition, CPA downregulated ER-stress and autophagy markers, including p-eIF2α/eIF2α, ATF4, Bip, CHOP, Beclin-1, Atg5, P62, and LC3B. Additionally, CPA influenced gut microbiota composition, increasing microbial diversity and restoring microbial balance in IBS-D rats. Linear discriminant analysis Effect Size (LEfSe) identified CPA-responsive taxa, further supporting CPA’s role in promoting a balanced and diverse gut microbiome. These experimental results suggested that CPA alleviates IBS-D by modulating the gut microbiota, regulating autophagy through the ER stress signaling pathway, and reinforcing colonic barrier integrity. This study highlights CPA as a promising therapeutic candidate for IBS-D.

## 1 Introduction

Irritable bowel syndrome (IBS) is a disorder of gut–brain interaction (DGB) caused by multiple factors, including the disruption of the brain-gut-microbiota axis (Black, 2021; Yadav et al., 2021; Black et al., 2024; Yuan et al., 2023; Wu et al., 2022). IBS affects approximately 10% of the global population, which results in annual socioeconomic losses amounting to billions of dollars (Lembo et al., 2022; Shah et al., 2022; Lovell and Ford, 2012; Oka et al., 2020). IBS can be classified into four types based on stool consistency: diarrhea-predominant (IBS-D), constipation-predominant (IBS-C), mixed (IBS-M), and unclassified (IBS-U) types, with IBS-D being the most common. IBS-D is characterized by recurrent diarrhea and abdominal pain (Sood et al., 2014).

Currently, standard treatments for IBS-D include antidiarrheals, antispasmodics, opioid receptor agonists, antidepressants, and probiotics (Ashburn and Gupta, 2006; Camilleri and Boeckxstaens, 2023). However, these treatments often have significant side effects and stability issues, impacting treatment outcomes and clinical applications (Nee and Lembo, 2021; Paine, 2021; Tack et al., 2006). Thus, treating IBS-D necessitates an individualized approach that combines medication, dietary adjustments, and lifestyle changes (Lembo et al., 2022; Shah et al., 2022; Chey et al., 2021; Wechsler and Shah, 2021; Wang et al., 2023; Camilleri and Boeckxstaens, 2017; Mozaffari et al., 2024). Given the efficacy and side effects of existing treatments, the search for new drugs to act as alternatives or supplements in clinical practice is of considerable practical importance (Camilleri and Boeckxstaens, 2023; Camilleri, 2015).

*Codonopsis pilosula*, a plant with a long history of use in both food and medicine, has been extensively used in traditional Chinese medicine for centuries due to its ability to improve multiple health-related parameters, making it one of the most important medicinal herbs (Meng et al., 2020; Luan et al., 2021; Zou et al., 2020). The National Health Commission of China has recently listed edible plants as substances used in food and traditional Chinese medicine. *Codonopsis pilosula* is commonly used as a principal or supplementary herb in traditional Chinese medicine. Modern medical research has progressively uncovered various pharmacological effects of *C. pilosula*, including its antibacterial, anti-inflammatory, and gut microbiota-regulating properties (Meng et al., 2020; Zou et al., 2020; Ruan et al., 2023). One of the primary components of *C. pilosula*, inulin-type fructan (*C. pilosula* polysaccharides, CPA), has demonstrated significant efficacy in treating intestinal diseases such as ulcerative colitis (Zhou et al., 2024a; Zhou et al., 2024b). CPA shows promise in this regard, offering a new treatment option for IBS-D patients due to its antibacterial, anti-inflammatory, and gut microbiota-regulating properties, which may help reduce side effects and improve treatment outcomes. Mechanistically, as an inulin-type fructan, CPA resists upper digestive degradation and reaches the colon intact, where it serves as a prebiotic substrate for gut microbiota. Its fermentation yields short-chain fatty acids (SCFAs) that modulate intestinal immune responses and barrier function. Meanwhile, emerging evidence indicates that inulin-type fructans can alleviate ER stress and influence autophagy in intestinal epithelial cells (Zhou et al., 2024b; Zhang et al., 2024). Given the crucial roles of gut microbiota dysbiosis and ER stress-induced autophagy in IBS-D, CPA likely exerts its therapeutic effects precisely through these pathways.

This study aims to provide theoretical and experimental foundations for treating IBS-D and developing new drugs by investigating the protective effect of CPA and its underlying mechanism. To explore this, we first conducted an analysis of GSE36701 dataset from the GEO to identify genes associated with IBS-D, and subsequently constructed an IBS-D rat animal model induced by Senna leaves combined with chronic unpredictable stimuli, as well as an LPS-induced NCM460 cell model to clarify the mechanism underlying CPA’s protective effect.

## 2 Materials and methods

### 2.1 Acquisition of GEO microarray data and WGCNA construction

The gene expression dataset GSE36701 was obtained from the GEO (https://www.ncbi.nlm.nih.gov/geo/). This dataset includes expression data from 221 samples, comprising a comparison between 27 diarrhea-predominant irritable bowel syndrome (IBS-D) subjects and 21 healthy volunteers (HVs). WGCNA is a widely used systems biology method for investigating the gene association patterns across samples, identifying gene modules with synergistic expression changes, and discovering potential biomarkers or therapeutic targets by analyzing the correlations between gene modules and phenotypic traits. In this study, WGCNA was performed using the “WGCNA” package in R. Specifically, hierarchical clustering of samples was conducted using the hclust function to identify and remove outliers. The pickSoftThreshold function was then employed to determine the optimal soft-thresholding power that ensures the network satisfies the scale-free topology criterion (scale-free R^2^ ≥ 0.9). Gene similarity was assessed based on the Topological Overlap Matrix (TOM), followed by the construction of a gene dendrogram using average linkage hierarchical clustering. Dynamic tree cutting algorithm (cutreeDynamic with parameters: deepSplit = 2, minModuleSize = 50) was applied to identify initial modules, which were subsequently merged using a TOM dissimilarity threshold of 0.25 to combine highly similar modules. Finally, module eigengenes (MEs) were calculated for each module, and their correlations with the target traits were evaluated to provide a basis for subsequent biomarker discovery.

### 2.2 Chemicals and reagents

CPA (3.6 kDa, inulin-type fructan, purity ≥98%): *C. pilosula* was collected from Shangdang, Changzhi, Shanxi. The roots were soaked in methanol and water, boiled, filtered, concentrated, and freeze-dried to obtain CPA powder. Senna Leaf Powder: Senna Leaves were purchased from Zexintang Pharmaceutical Co., Ltd., Bozhou, Anhui Province, with product number 10002765021. Senna leaves were soaked, boiled, filtered, concentrated, and freeze-dried to obtain powder. Berberine Hydrochloride: Purchased from Northeast Pharmaceutical Group Co., Ltd., with the national drug approval numbers H21022453 and batch number 2202002.

### 2.3 Animal model establishment

Eight-week-old male Sprague Dawley rats (SPF-grade), weighing approximately 180–200 g, were purchased from SPF (Beijing) Biotechnology Co., Ltd. (License number: SCXK (Beijing) 2019-0010). The rats were housed under controlled conditions with a temperature of 22 °C ± 2 °C, a humidity of 65% ± 5%, and a 12-h light-dark cycle (light from 8:00 to 20:00). They had free access to standard food and water. The animal experimental scheme was strictly implemented in accordance with the ethical code of Animal Care and Use Committee of Shanxi Medical University and the Guidelines for The Care and Use of Laboratory Animals (with approval number: SYXK (Jing) 2017-0005). All efforts were made to minimize animal suffering. After a 1-week acclimatization period, some rats were randomly selected as the control group. In contrast, the remaining rats were subjected to chronic, unpredictable stress to induce an IBS-D model.

The IBS-D rat model was established by administering senna leaf powder orally at a dose of 6 g/kg/day, with chronic unpredictable stimuli for 14 consecutive days. The success of the model and treatment was evaluated using Bristol stool grading and Abdominal Withdrawal Reflex.

### 2.4 Experimental animal grouping and management

A total of 60 SPF-grade male rats were used and randomly assigned to six groups (n = 10 per group). Male rats were selected to avoid variability introduced by the estrous cycle in females, which can confound behavioral, biochemical, and histological outcomes and reduce statistical power and reproducibility. Berberine was chosen as the positive control at a dose of 0.06 g/kg/day (p.o.) based on previous pharmacological studies in IBS-D rat models (Yu et al., 2019; Lu et al., 2021) and our preliminary experiments. The successfully modeled rats were randomly assigned to five groups: berberine (0.06 g kg^−1^ day^−1^), model group, CPA-L (low dose of CPA, 0.03 g kg^−1^day^−1^), CPA-M (medium dose of CPA, 0.06 g kg^−1^day^−1^), and CPA-H (high dose of CPA, 0.09 g kg^−1^day^−1^). Thymus index (mg/g) = Thymus weight (mg)/Body weight (g)×100. Spleen index (mg/g) = Spleen weight (mg)/Body weight (g) ×100. The experimental design is shown in the following figure (Figure 1A).

**FIGURE 1:**
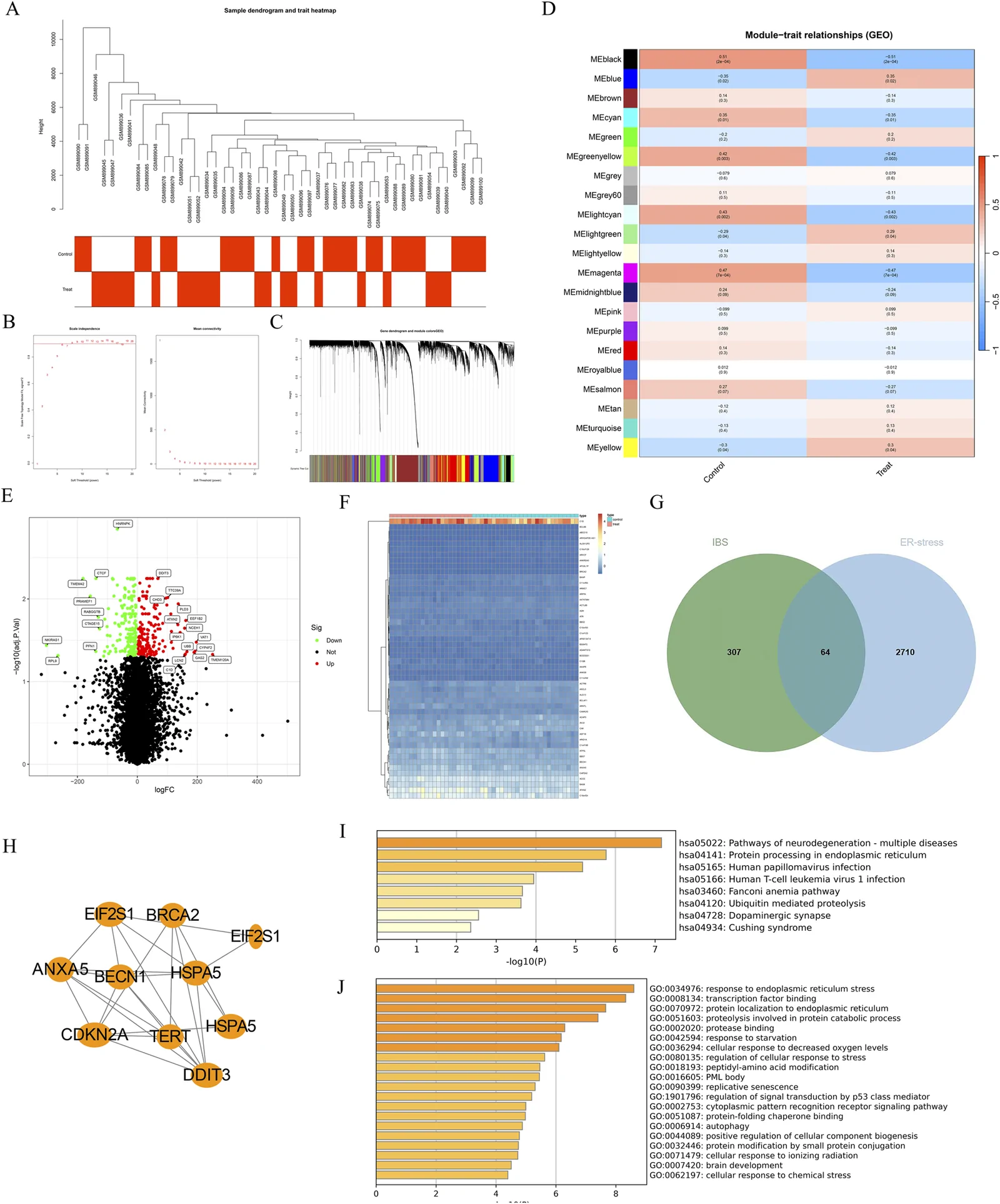
Identification of hub genes related to IBS in the weighted co-expression network. **(A)** Clustered heatmap of sample features. **(B)** Analysis of soft-thresholding power for scale-free topology fit index (left) and mean connectivity (right). The red line indicates the selected power based on the correlation coefficient. **(C)** Cluster dendrogram of co-expressed genes. **(D)** Heatmap of gene expression patterns. **(E)** Volcano plot showing DEGs in the GSE36701 dataset. **(F)** Heatmap displaying the top 100 upregulated and top 100 downregulated genes in IBS versus control groups from the GSE36701 dataset. **(G)** Venn diagram illustrating the intersection of genes from the GSE36701-WGCNA analysis and ER stress-related genes. **(H)** Top 10 hub genes identified by the Maximal Clique Centrality (MCC) algorithm from Cytohubba. **(I)** KEGG pathway enrichment analysis results. **(J)** GO functional enrichment analysis results.

### 2.5 Cell model establishment

Culture medium: DMEM supplemented with 10% FBS. The IBS-D cell model was created by stimulating normal human colon cells (NCM460) with lipopolysaccharide (LPS, 1 μg mL^−1^, purchased from Solarbio, L88800). Initially, the cells were seeded into a 96-well plate, and a CCK8 assay (purchased from Boster, AR1199) was used to determine the proliferation activity of CPA in the cells, establishing the optimal CPA concentration. Once the optimal CPA concentration was established, NCM460 cells were seeded into a 6-well plate and divided into five groups: control, LPS, CPA-L, CPA-M, and CPA-H. The control group received complete medium containing serum in each well, while the other groups were treated with serum-free 1,640 medium containing LPS (1 μg mL^−1^). After 24 h, the control and LPS groups received complete medium with serum, while the remaining groups were treated with a complete medium containing the respective CPA doses for another 24 h. Following the culture period, the medium was discarded, and the cells were collected for analysis.

### 2.6 Cytokine analysis

The expression levels of the inflammatory cytokines TNF-α and IL-6, the anti-inflammatory cytokines IL-4, IL-10, and IL-13, the pain-related factor 5-HT, and the analgesic factor NP-Y were quantified using enzyme-linked immunosorbent assay (ELISA) kits (Jiangsu Meimian Industrial Co., Ltd, Jiangsu, China).

### 2.7 HE stainings

Fresh rat colon tissues were rinsed in saline and then fixed in 4% paraformaldehyde. The tissues were dehydrated, embedded in paraffin, and sectioned into thin slices. These sections were deparaffinized to water, and frozen sections were warmed for fixation. After pretreatment, the sections were sequentially stained with hematoxylin and eosin. The stained sections were then dehydrated, mounted, and observed under an upright microscope, with images captured for analysis. The nuclei appeared blue, while the cytoplasm seemed to be red.

### 2.8 AB-PAS staining

Prepared paraffin sections were deparaffinized to water, and frozen sections were warmed for fixation. Following pretreatment, the sections were stained sequentially using AB-PAS Staining Solutions C, B, and A. The stained sections were dehydrated, mounted, and observed under an upright microscope. Acidic mucins appeared blue, glycogen and neutral mucins appeared purplish-red, and mixed mucins appeared purplish-blue or violet.

### 2.9 Immunohistochemistry (IHC)

Prepared paraffin sections were deparaffinized to water. After antigen retrieval, endogenous peroxidase activity in the sections was blocked, and the sections were then subjected to serum blocking and incubation with primary and secondary antibodies. DAB was used for color development, followed by nuclear counterstaining. After dehydration and mounting, the sections were observed under a bright-field microscope. Hematoxylin-stained nuclei appeared blue, while positive DAB staining was seen as brownish-yellow.

### 2.10 Immunofluorescence (IF)

Prepared paraffin tissues and NCM460 cell sections were deparaffinized to water. After antigen retrieval, the sections were blocked with serum and incubated with primary and secondary antibodies. DAPI was used for nuclear counterstaining. Endogenous fluorescence was quenched, and the sections were then mounted. In the DAPI channel, nuclei appeared blue; in the CY3 channel, positive staining seemed red.

### 2.11 CCK-8 assay

NCM460 cells were seeded into 96-well plates and cultured for 24 h. The cells were then treated with various concentrations of CPA, followed by the addition of CCK8 reagent. After 2 hours of incubation, the absorbance of each well was measured at 450 nm.

### 2.12 qRT-PCR

Total RNA from NCM460 cells was extracted and reverse transcribed using the M5 Super Plus qPCR RT kit (Mei5 Biotechnology, Beijing, China), following the manufacturer’s instructions. Gene expression changes were measured using the Light Cycler system with 2x M5 HiPer SYBR Premix EsTaq (with Tli RNaseH). β-actin was used as an internal control. The primer sequences were presented in Table 1.

**TABLE 1:** Primer sequences.

Gene	Direction	Sequences	PCR products (bp)
TNF-α	Forward	TGG​CGT​GGA​GCT​GAG​AGA​TAA​C	76
	Reverse	GAA​GAG​GAC​CTG​GGA​GTA​GAT​GAG	76
IL-1β	Forward	CAG​TGG​CAA​TGA​GGA​TGA​CTT​GTT​C	129
	Reverse	CTG​TAG​TGG​TGG​TCG​GAG​ATT​CG	129
IL-6	Forward	TGG​TGT​TGC​CTG​CTG​CCT​TC	139
	Reverse	GCT​GAG​ATG​CCG​TCG​AGG​ATG	139
IL-10	Forward	GGG​TTG​CCA​AGC​CTT​GTC​TGA​G	86
	Reverse	CCT​TGA​TGT​CTG​GGT​CTT​GGT​TCT​C	86
IL-13	Forward	CAT​GTA​CTG​TGC​AGC​CCT​GGA​ATC	88
	Reverse	GCA​GAA​TCC​GCT​CAG​CAT​CCT​C	88

### 2.13 Western blots

Proteins were extracted using RIPA buffer with protease and phosphatase inhibitors. Protein concentrations were measured by BCA assay and then normalized. Proteins were separated by 10% SDS-PAGE and transferred to PVDF membranes, which were then blocked with 5% BSA. Membranes were incubated with primary antibodies overnight at 4 °C, followed by secondary antibody incubation for 1 h at room temperature. Detection was performed using ECL substrate and imaging. The primary and secondary antibodies, along with their dilutions, were listed as follows: P-eIF2α (1:5,000, 28740-1-AP, Proteintech), eIF2α (1:1,000, 11170-1-AP, Proteintech), eIF2α (1:500, A0764, ABclonal), ATF4 (1:750, 10835-1-AP, Proteintech), CHOP (1:1,500, 15204-1-AP), GRP78/BIP (1:6,000, 11587-1-AP), ATG5 (1:4,500, 10181-2-AP), Beclin-1 (1:5,000, 11306-1-AP, Proteintech), P62 (1:1,000, A19700, ABclonal), LC3B (YN5524, 1:1,500, Immunoway), β-actin (1:7,000, 20536-1-AP, Proteintech). HRP-Conjugated AffiniPure Goat Anti-Rabbit IgG (H + L) (1:10,000, BA1054, Boster). For antibody specificity verification, the ATF4 and CHOP antibodies were validated by the manufacturer (Proteintech) using ER stress-positive cell lysates and gene knockout negative controls. Proteintech ATF4 and CHOP antibodies have also been successfully applied in published studies investigating intestinal ER stress and intestinal injury models (Liu et al., 2024; Zhang et al., 2025).

### 2.14 Gut microbiota

Total genomic DNA was extracted using the Mag-bind Soil DNA Kit and assessed for quality and concentration. The V3-V4 region of the 16S rRNA gene was PCR-amplified with barcoded primers and high-fidelity DNA polymerase. PCR products were gel-purified, quantified, and pooled for library construction using the Illumina TruSeq Nano kit. Libraries were validated via Agilent Bioanalyzer and Promega QuantiFluor before sequencing on an Illumina platform. Paired-end reads were processed in QIIME2 (v.latest) with adapter removal (cutadapt), quality filtering, denoising, merging, and chimera removal (DADA2), generating ASVs and abundance tables. Taxonomic assignment was done using the classify-sklearn method with the SILVA database (v138). Alpha and beta diversity were assessed, and community composition differences were analyzed with LEfSe and statistical tests in STAMP.

### 2.15 Data analysis

Data was plotted and analyzed using GraphPad Prism 10.0. All data were presented as mean ± SD. One-way or two-way ANOVA was used to determine significant differences between groups with multiple comparisons and the least significant difference test. A p-value of <0.05 was considered statistically significant.

## 3 Results

### 3.1 Identification of hub genes associated with IBS via WGCNA

WGCNA was a widely used method for identifying gene modules associated with external traits. In this study, WGCNA was employed to analyze genes in IBS samples. By constructing a scale-free gene co-expression network, the study aimed to identify hub genes. The analysis utilized 48 samples from the GSE36701 dataset. After quality control, a normalized expression matrix containing 369 genes was obtained (Figure 1A). A soft-thresholding power of β = 8 (scale-free R^2^ = 0.85) was selected to construct the scale-free network (Figure 1B). Through average linkage hierarchical clustering, 21 modules were finally identified (Figure 1C). Pearson correlation analysis revealed that the MEblack module, which contains 178 genes, exhibited a significantly positive correlation with IBS (r = 0.74, p = 5e-20) (Figure 1D). Furthermore, within the MEblack module, a significant positive correlation was also observed between gene significance (GS) and module eigengene (ME) (r = 0.86, p = 1e-200). A volcano plot was generated (Figure 1E); genes with logFc >0 and adj <0.05 were defined as upregulated genes, while those with logFc <0 and adj <0.05 were downregulated genes. This analysis identified 168 upregulated genes and 201 downregulated genes. A heatmap was used to visualize the top 200 differentially expressed genes (DEGs) (Figure 1F). A total of 176 genes related to endoplasmic reticulum stress were retrieved from the GeneCards database (https://www.genecards.org/) and Online Mendelian Inheritance in Man (OMIM) database (https://www.omim.org/). Using an online Venn diagram (Figure 1G), 64 intersecting genes associated with both IBS and ER stress were obtained. Subsequently, functional enrichment analysis was performed on these 64 key genes to explore their potential molecular functions. Gene Ontology (GO) enrichment analysis showed that these genes were mainly involved in the response to ER stress, transcription factor binding, and protein localization to the ER (Figure 1J). Kyoto Encyclopedia of Genes and Genomes (KEGG) analysis linked these genes to signaling pathways including “protein processing in endoplasmic reticulum”, “neurodegenerative disease pathways”, and “ubiquitin-mediated proteolysis” (Figure 1I). To further clarify the relationships among these key genes, a protein-protein interaction (PPI) network was constructed based on the STRING database and visualized using Cytoscape software. The top 10 hub genes were identified (Figure 1H). Among them, HSPA5 and DDID3 were both core targets of the endoplasmic reticulum stress signaling pathway. Based on above, we hypothesized that IBS-D is closely associated with the ER-stress signaling pathway.

### 3.2 CPA improves the macroscopic representation in rats

The results demonstrated the impact of CPA treatment on macroscopic features in senna-leaf-induced IBS-D rats (Figure 2). Specifically, the model group exhibited a significant decrease in body weight over the 30-day experimental period compared to the control group, while treatment groups experienced less pronounced decreases (Figure 2C). Organ indices revealed a reduction in thymus index and spleen index in the model group compared to the control, with treatment groups showing improvement toward control levels (Figures 2E,F). The model group also showed significantly elevated abdominal withdrawal reflex (AWR) scores across all tested pressure levels compared to the control. Treatment with berberine, CPA-L, CPA-M, and CPA-H significantly reduced AWR scores relative to the model group at each pressure level (Figure 2B). The Bristol stool scale scores were higher in the model group than those in the control group. In contrast, treatment groups showed scores significantly reduced compared to the model group (Figure 2D). Across all measured parameters, treatment with berberine and CPA demonstrated significant improvements compared to those in the model group, with statistical significance indicated by *P <* 0.05 and *P <* 0.01.

**FIGURE 2:**
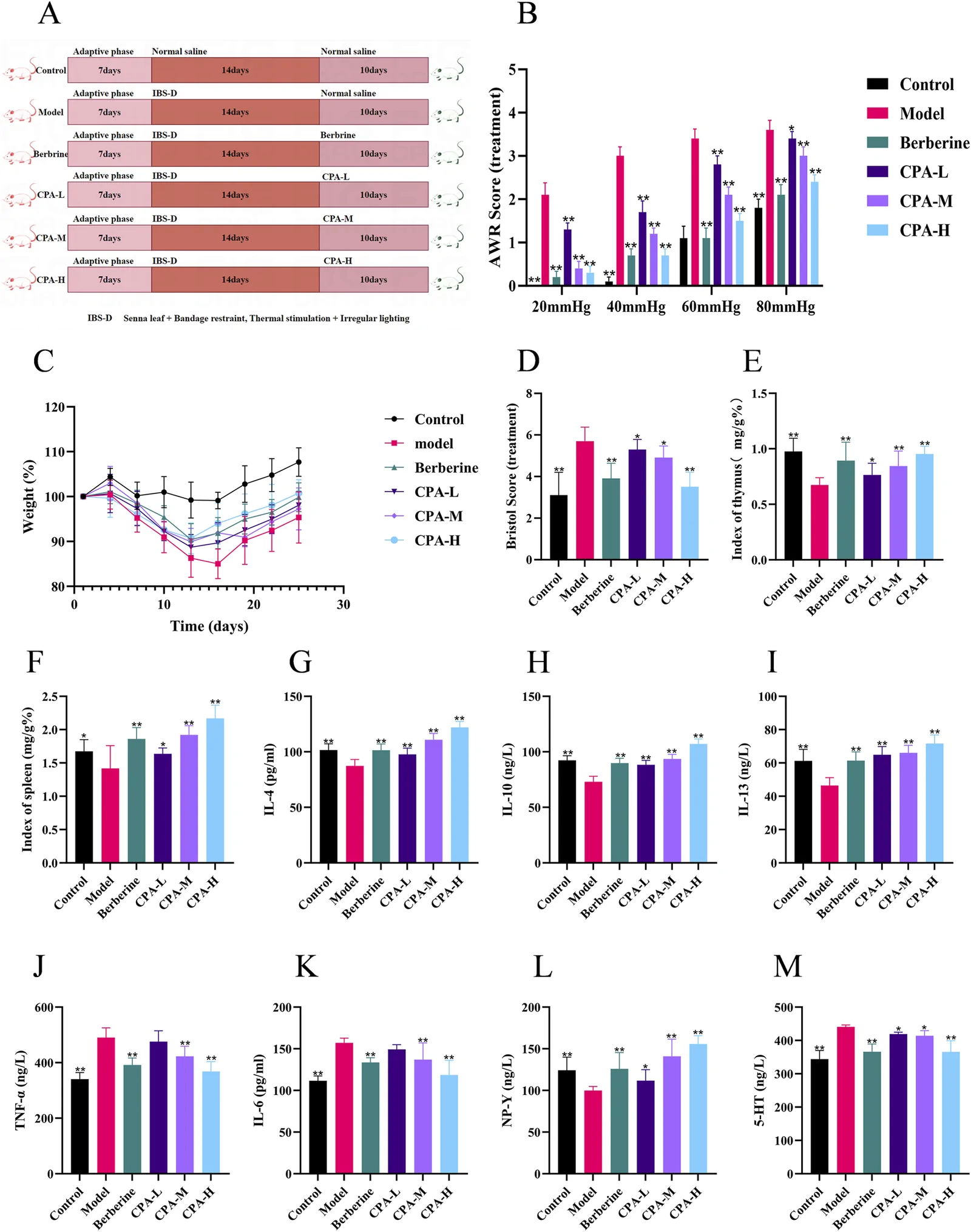
Effects of different treatments on irritable bowel syndrome (IBS)-related parameters, cytokine levels, and neurotransmitter levels in rats. **(A)** Experimental design flowchart. **(B)** Abdominal withdrawal reflex (AWR) score. **(C)** Changes in mouse body weight. **(D)** Bristol stool score. **(E)** Thymus index. **(F)** Spleen index. **(G)** Interleukin-4 (IL-4) level. **(H)** Interleukin-10 (IL-10) level. **(I)** Interleukin-13 (IL-13) level. **(J)** Tumor necrosis factor-α (TNF-α) level. **(K)** Interleukin-6 (IL-6) level. **(L)** Neuropeptide Y (NP-Y) level. **(M)** 5-Hydroxytryptamine (5-HT) level. Data are expressed as mean ± SD. Compared to the model group, * indicates *P <* 0.05, and ** indicates *P <* 0.01. n = 10 per group.

### 3.3 CPA improves inflammatory and pain state in rats

Compared to the control group, the model group exhibited a significant decrease in the levels of IL-4 (Figure 2G), IL-10 (Figure 2H), IL-13 (Figure 2I), NP-Y (Figure 2L), and 5-HT (Figure 2M) (*P <* 0.01), whereas the levels of TNF-α (Figure 2J) and IL-6 (Figure 2K) were significantly promoted (*P <* 0.01). Compared to the model group, the berberine group showed significantly increased levels of IL-4 (Figure 2G), IL-10 (Figure 2H), IL-13 (Figure 2I), and NP-Y (Figure 2L) (*P <* 0.01), and significantly decreased levels of TNF-α (Figure 2J) and IL-6 (Figure 2K), and 5-HT (Figure 2M) (*P <* 0.01). Compared to the model group, the CPA-L, CPA-M, and CPA-H groups all showed significant increases in the levels of IL-4 (Figure 2G, *P <* 0.01), IL-10 (Figure 2H, CPA-M and CPA-H groups, *P <* 0.01), IL-13 (Figure 2I, *P <* 0.01), NP-Y (Figure 2L, CPA-M and CPA-H groups, *P <* 0.01), and significant decreases in the levels of TNF-α (Figure 2J, CPA-M and CPA-H groups, *P <* 0.01), IL-6 (Figure 2K, CPA-M and CPA-H groups, *P <* 0.01) and 5-HT (Figure 2M, CPA-L group, *P <* 0.05, CPA-M and CPA-H groups, *P <* 0.01).

### 3.4 CPA improves colonic structure in rats

In the control group, the colonic tissue exhibited a regular structure with a transparent mucosal layer and an orderly arrangement of cells in the lamina propria. In contrast, the tissue structure in the model group demonstrated marked changes, including disruption of the colonic mucosal layer, significant inflammatory cell infiltration, as indicated by the arrows, and a disorganized arrangement of cells in the lamina propria (Figure 3A). The berberine-treated group displayed reduced levels of inflammatory cell infiltration, and the mucosal layer structure was partially recovered. The colonic tissue structure of the CPA-L group resembled that of the berberine group, showing a decrease in inflammatory cell infiltration compared to that in the model group. Notably, the colonic tissue structure in the CPA-H group closely resembled the normal state, with inflammatory cell infiltration nearly absent.

**FIGURE 3:**
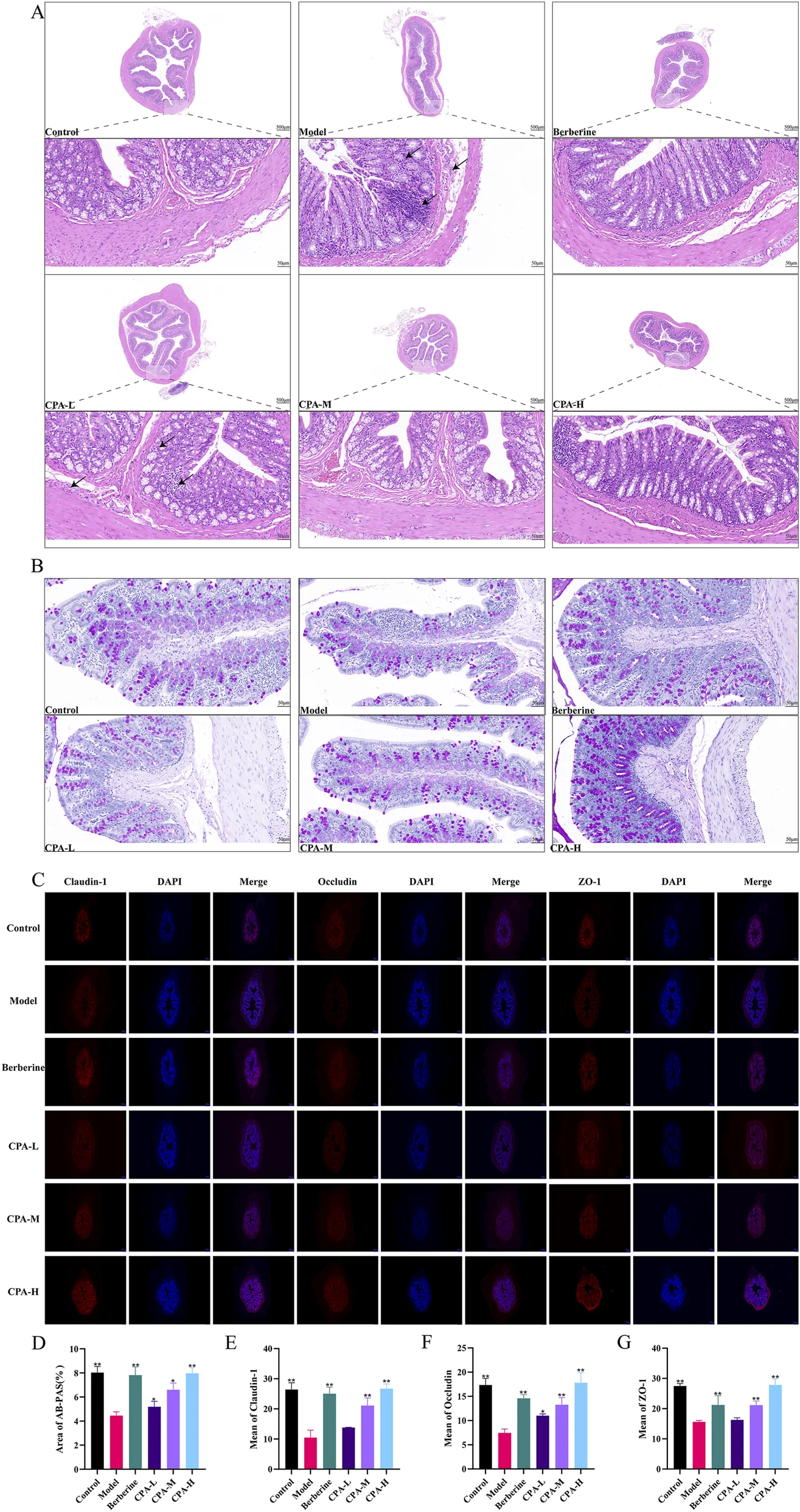
Effects of different treatments on colonic histopathology, mucus layer, and tight junction protein expression. **(A)** Representative images of Hematoxylin and eosin (H&E) staining of colonic tissue. The top row shows low-magnification images of entire colonic cross-sections; the bottom row shows high-magnification views of the corresponding boxes. Arrows highlight infiltration of inflammatory cells. Scale bars: 500 μm (top row), 50 μm (bottom row). **(B)** Representative images of Alcian blue-periodic acid-Schiff (AB-PAS) staining of colonic tissue. Scale bar: 50 μm. **(C)** Quantification of the AB-PAS-stained area (%). **(D)** Representative immunofluorescence staining of Claudin-1, Occludin, and ZO-1 (red) with DAPI counterstaining (blue) in colonic tissue. Merged images show the co-localization. **(E–G)** Quantitative analysis of the mean fluorescence intensity for **(E)** Claudin-1, **(F)** Occludin, and **(G)** ZO-1. Data are presented as mean ± SD. *P < 0.05 and **P < 0.01 compared to model group. n = 3 Per group.

### 3.5 CPA improves colonic barrier in rats

The histological observation of colonic tissue in each group revealed that the mucus secretion in the model group was significantly lower than that in the control group, accompanied by a marked reduction in the expression of tight junction proteins Claudin-1, Occludin, and ZO-1 (Figures 3B,C). Compared to the model group, both the berberine-treated group and all CPA-treated groups showed an increase in mucus secretion and a significant upregulation in the expression of Claudin-1, Occludin, and ZO-1 (Figures 3D–G, *P <* 0.01).

### 3.6 CPA regulates ER stress-induced autophagy in rat colonic tissue

Compared to the control group, the model group showed significant increases in protein expression levels of Bip, p62, ATF4, Beclin-1, Atg5, CHOP, p-eIF2α, and LC3II/LC3I ratio (*P <* 0.05 or *P <* 0.01). The related protein expression in the berberine group was significantly reduced compared to that in the model group. In the CPA-L group, the expressions of these proteins were also significantly decreased. In the CPA-M and CPA-H groups, the protein expression of these markers was further reduced, especially in the CPA-H group (Figure 4).

**FIGURE 4:**
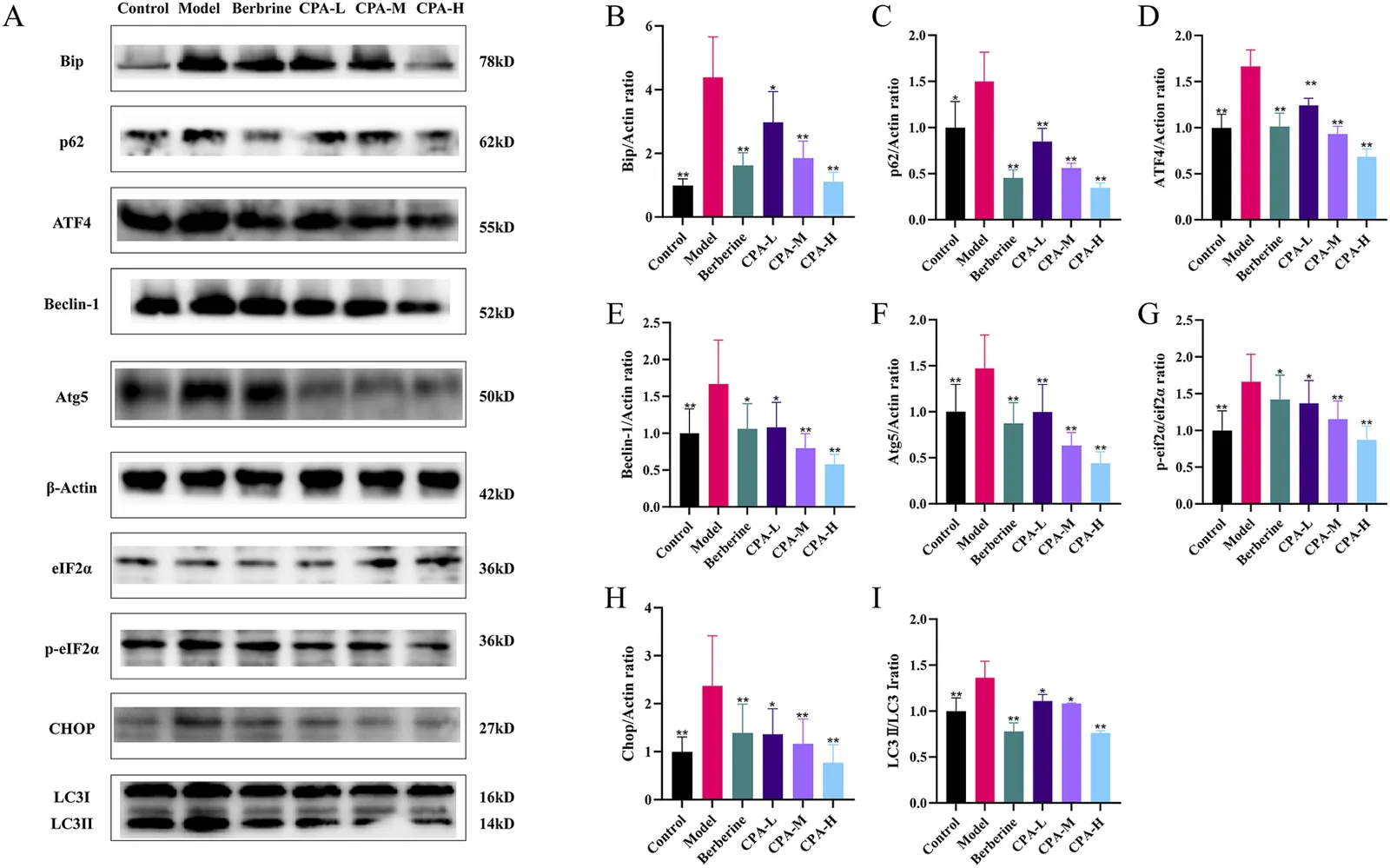
Protein expression analysis of ER stress and autophagy markers by Western blotting. **(A)** Representative Western blot images showing the protein levels of Bip, p62, ATF4, Beclin-1, Atg5, β-actin, eIF2α, p-eIF2α, CHOP, LC3I and LC3II. **(B–I)** Quantification of relative protein expression. Protein levels were normalized to β-actin, except for the LC3II/LC3I ratio, a ratio between two specific protein bands. Data are presented as mean ± SD. *P < 0.05 and **P < 0.01 compared to the model group. n = 3 Per group.

Compared to that in the model group, the percentage of the stained area of Beclin-1, Atg5, LC3B, and p62 was significantly reduced in both the berberine treatment group and all the CPA groups (*P <* 0.05 or *P <* 0.01) (Figure 5A). The expression of these proteins showed a dose-dependent decrease in the CPA groups, with the CPA-H group showing expression levels closest to the control group (Figures 5B–E).

**FIGURE 5:**
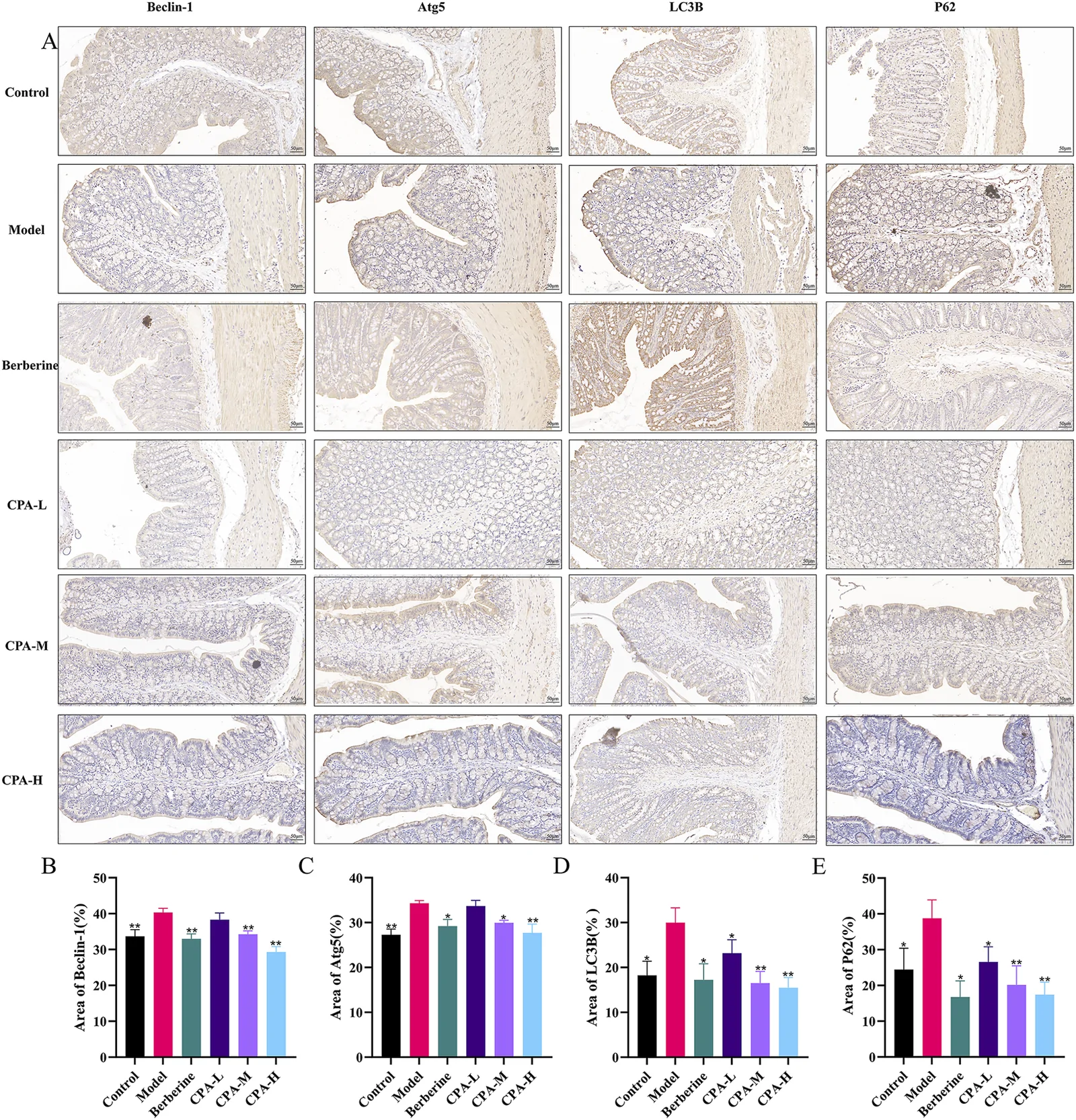
Immunohistochemical staining of autophagy-related proteins in colonic tissue. **(A)** Representative immunohistochemistry images showing the expression of Beclin-1, Atg5, LC3B, and p62 in colonic tissue from each group. Scale bars represent 100 μm. **(B–E)** Quantifying the percentage of stained area of Beclin-1, Atg5, LC3B, and p62, respectively. Data are presented as mean ± SD. *P < 0.05 and **P < 0.01 compared to the model group. n = 3 Per group.

### 3.7 CPA improves NCM460 cell viability and inflammation

At low doses (0–200 μg/mL) of CPA, the cell viability was not significantly different compared to the control group. The cell viability was increased considerably when the CPA concentration was increased to 400 μg/mL or higher (*P <* 0.01; Figure 6A). Compared to the control group, LPS stimulation led to a significant increase in the mRNA expression of TNF-α, IL-1β, and IL-6, while that of IL-10 and IL-13 was decreased (*P <* 0.01). Compared to the LPS group, the mRNA expression of TNF-α, IL-1β, and IL-6 were significantly decreased in the CPA-L group, while that of IL-10 and IL-13 was increased (*P <* 0.05 or *P <* 0.01). In the CPA-H group, the expression levels of pro-inflammatory cytokines were close to those of the control group. In contrast, the expression levels of anti-inflammatory cytokines were significantly higher than those of the LPS group (*P <* 0.01; Figures 6B–F).

**FIGURE 6:**
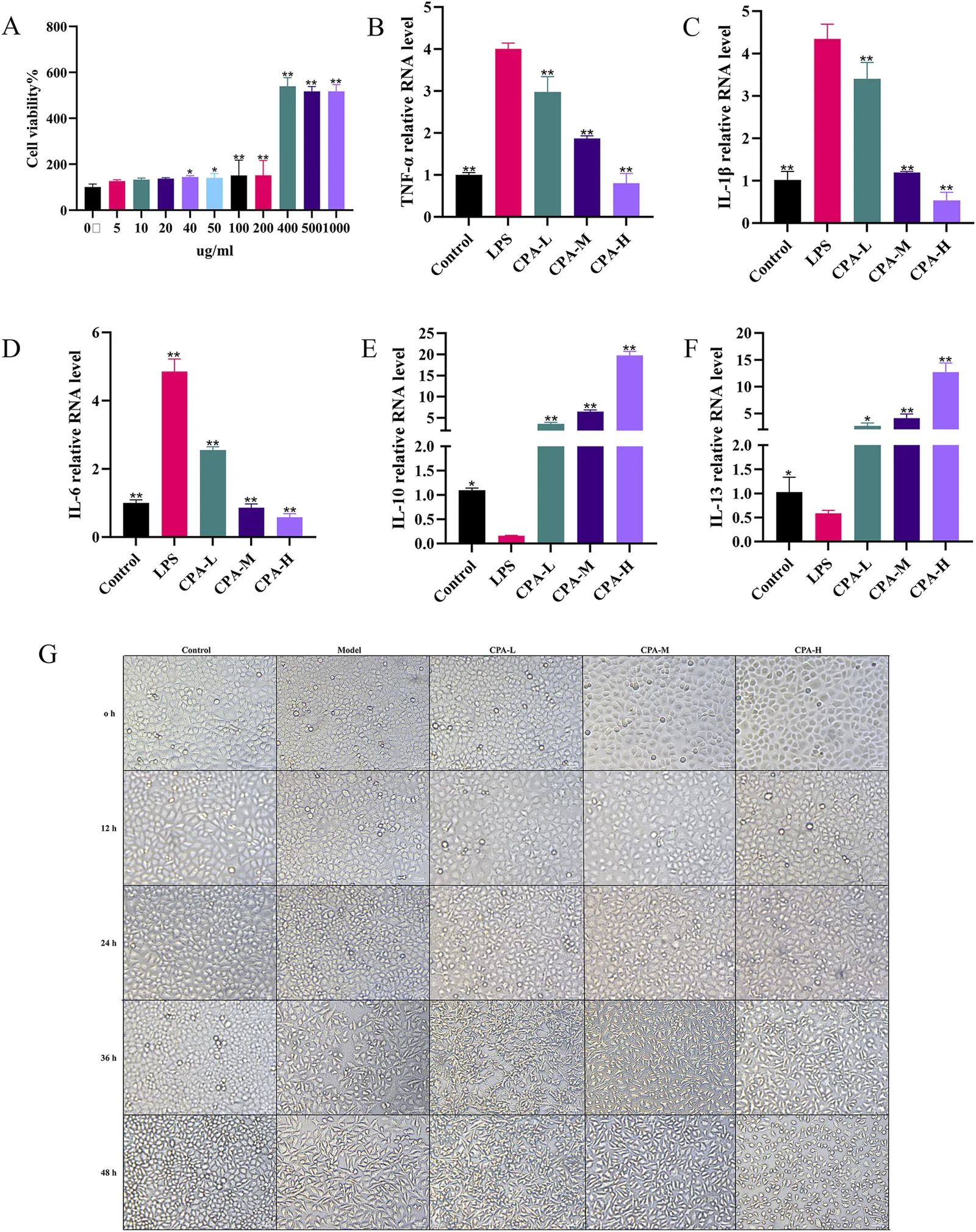
Effects of CPA on cell viability, inflammatory response, and morphology in NCM460 cells. **(A)** Cell viability of NCM460 cells treated with different concentrations of CPA. **(B–F)** mRNA expression levels of TNF-α, IL-1β, IL-6, IL-10, and IL-13 in cells stimulated with LPS and then treated with different doses of CPA. **(G)** Representative bright-field images showing morphological changes of NCM460 cells over time (0, 12, 24, 36, 48 h) under different treatment conditions (Control, Model, CPA-L, CPA-M, CPA-H). Scale bars: 50 μm. Data in panels **(A–F)** are presented as mean ± SD (n = 3). *P < 0.05 and **P < 0.01 compared to the LPS group. n = 3 Per group.

### 3.8 CPA improves NCM460 cell growth

In the model group, cells exhibited irregular morphology, increased spacing, and decreased density, and these abnormalities worsened over time. The CPA-L group showed slight improvement, with better morphology and increased density. The CPA-M group had more noticeable improvement, with near-normal morphology and higher density. The CPA-H group closely resembled the control group, with tightly arranged cells and regular morphology (Figure 6G).

### 3.9 CPA improves NCM460 cell barrier

Compared to the LPS group, the mean fluorescence intensity of Claudin-1, Occludin, and ZO-1 was significantly increased in the berberine group and all CPA-treated groups (*P <* 0.05 or *P <* 0.01). The enhancement was more significant in the CPA-M and CPA-H groups, and the protein expression levels in the CPA-H group were closest to the control group (Figure 7).

**FIGURE 7:**
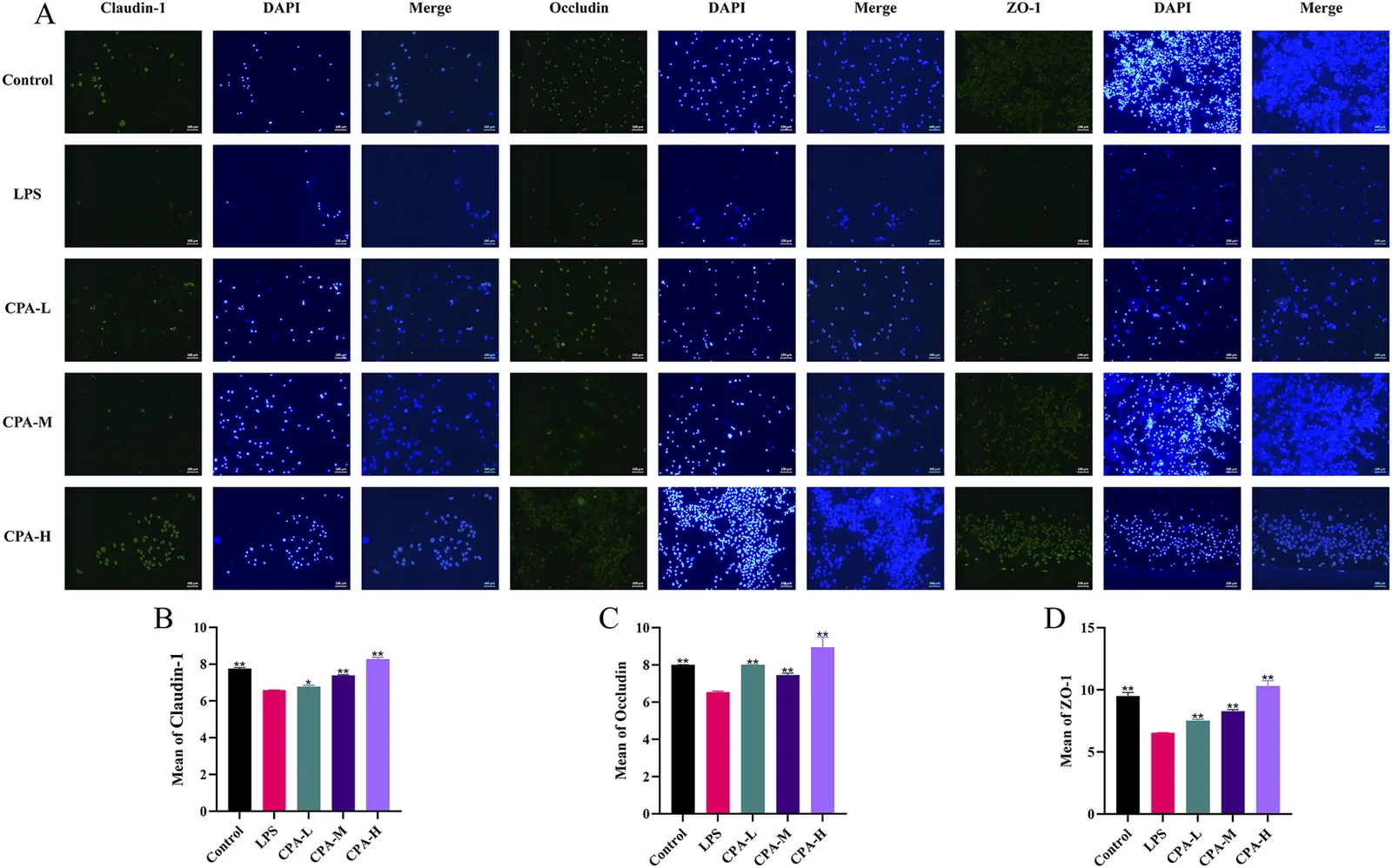
Immunofluorescence staining of tight junction proteins in NCM460 cells. **(A)** Representative immunofluorescence images showing the expression of Claudin-1, Occludin, and ZO-1 (green) and DAPI counterstaining (blue) in NCM460 cells from different treatment groups. Merged images show the co-localization of tight junction proteins with cell nuclei. **(B–D)** Quantifying the mean fluorescence intensity of Claudin-1, Occludin, and ZO-1, respectively. Data are presented as mean ± SD. *P < 0.05 and **P < 0.01 compared to the LPS group. n = 3 Per group.

### 3.10 CPA regulates endoplasmic reticulum stress in NCM460 cells

Compared to the LPS group, the expression of Bip, ATF4, p-eIF2α/eIF2α ratio, and CHOP was significantly reduced in the berberine group and CPA-treated groups (*P <* 0.05 or *P <* 0.01). In the CPA groups, the expression of these makers showed a dose-dependent decrease, with the expression levels in the CPA-H group being closest to the control group (Figure 8).

**FIGURE 8:**
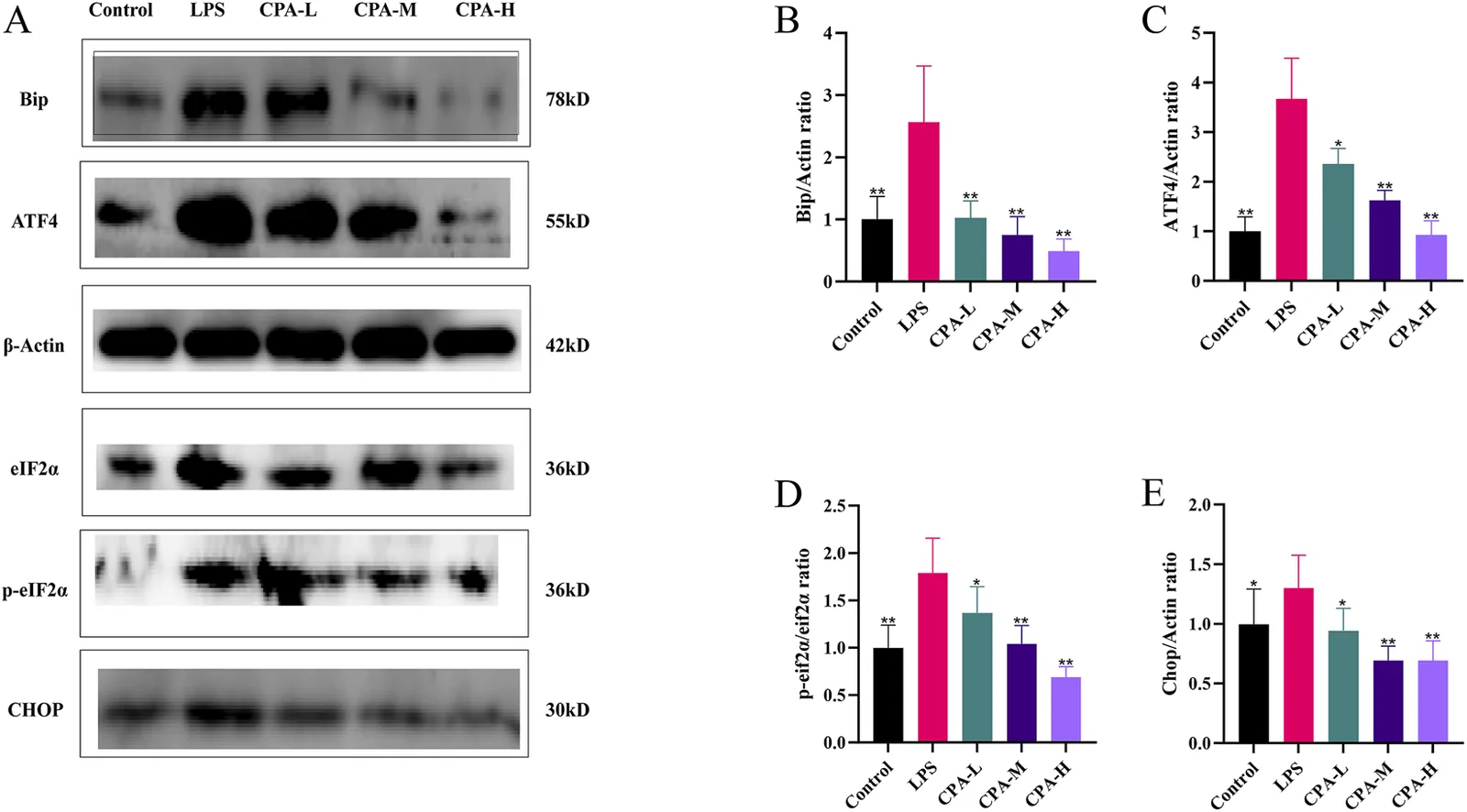
Protein expression analysis of ER stress markers by Western blotting in NCM460 cells. **(A)** Representative Western blot images showing the protein levels of Bip, ATF4, β-actin, eIF2α, p-eIF2α, and CHOP in NCM460 cells from different treatment groups. **(B–E)** Quantification of relative protein expression. Protein levels were normalized to β-actin, except for the p-eIF2α/eIF2α ratio. Data are presented as mean ± SD. *P < 0.05 and **P < 0.01 compared to the LPS group. n = 3 Per group.

### 3.11 CPA modulates gut microbiota structure and enhances community evenness

The sequencing depth for all samples was sufficient, as indicated by rarefaction curves reaching a plateau (Figure 9A). Analysis of amplicon sequence variants (ASVs) using a Venn diagram (Figure 9B) revealed a core microbiome of 1,046 shared ASVs across all treatment groups. The control, model, berberine, CPA-L, CPA-M, and CPA-H groups displayed distinct compositions, with 694, 341, 413, 273, 368, and 575 unique ASVs, respectively. Evaluation of alpha diversity demonstrated significant differences in microbial richness, with the control group exhibiting significantly higher observed ASVs and Chao1 indices (*P <* 0.01) than all other groups. In contrast, the model group showed the lowest observed ASVs and Chao1 indices (Figures 9C,D). Furthermore, the Shannon diversity index was relatively elevated in the CPA-M group (Figure 9E), suggesting a more even species distribution. However, Simpson’s index did not reveal significant intergroup differences (Figure 9F). Principal Coordinates Analysis (PCoA) (Figures 9G,H) separated the control and model groups, indicating substantial differences in their overall microbial community structures. Notably, the berberine and CPA-M groups exhibited partial overlap with the control group on the PCoA plots, suggesting some structural similarities.

**FIGURE 9:**
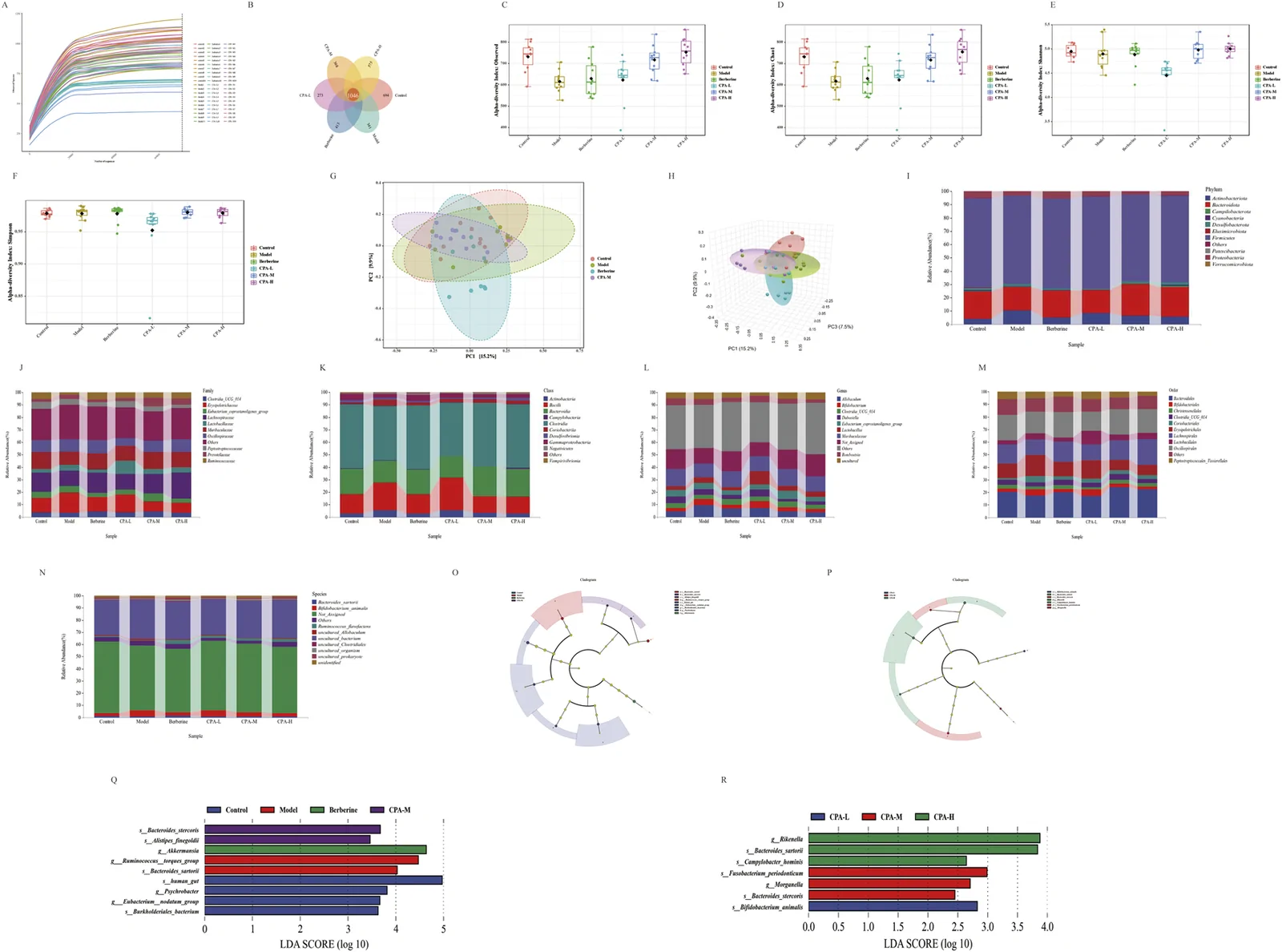
Comprehensive analysis of the gut microbiota in different treatment groups. **(A)** Rarefaction curves showing the relationship between the observed number of ASVs and the sequencing depth. **(B)** Venn diagram illustrating the number of shared and unique ASVs among groups. **(C–F)** Box plots of alpha diversity indices: **(C)** Observed ASVs, **(D)** Chao1, **(E)** Shannon, and **(F)** Simpson. **(G)** PCoA plot based on Bray-Curtis dissimilarity. **(H)** 3D PCoA plot visualizing the clustering of different groups. **(I)** Relative abundance of bacterial taxa at the phylum level. **(J)** Relative abundance at the family level. **(K)** Relative abundance at the class level. **(L)** Relative abundance at the genus level. **(M)** Relative abundance at the order level. **(N)** Relative abundance at the species level. **(O,Q)** Cladograms showing the phylogenetic distribution of differentially abundant microbes. **(P,R)** Linear Discriminant Analysis (LDA) scores for taxa with significant differences between groups. Data are presented as results of n = 10 rats per group. Statistical significance (*P* < 0.01 compared to the model group) for diversity metrics is specified in the detailed results.

### 3.12 CPA modulates gut microbiota relative abundance at different levels

At the phylum level (Figure 9I), *Firmicutes* and *Bacteroidota* were the dominant phyla across all groups, with *Firmicutes* showing an increase and *Bacteroidota* a decrease in the model group compared to the control group. The CPA-M and CPA-H groups partially reversed these changes, with a slight rise in *Bacteroidota* and a reduction in Firmicutes. *Actinobacteriota* was more prominent in the CPA-M and CPA-H groups than in other treatments.

At the class level (Figure 9K), Clostridia, *Bacteroidia*, and *Bacilli* were the most abundant classes. The model group showed an increase in Clostridia and a reduction in *Bacteroidia*, changes that were partially mitigated by CPA treatments. The CPA-L and CPA-M groups exhibited a higher abundance of *Clostridia* than other groups. At the order level (Figure 9M), *Bacteroidales*, *Clostridiales*, and *Lactobacillales* were predominant across all groups. The model group showed an enrichment in *Clostridiales* and a decrease in *Bacteroidales*, trends that were reversed in CPA-treated groups, particularly CPA-L and CPA-M, which exhibited a significant increase in *Clostridiales*.

At the family level (Figure 9J), the dominant families included *Lachnospiraceae*, *Ruminococcaceae*, and *Muribaculaceae*. The model group showed an enrichment of *Lachnospiraceae* and *Ruminococcaceae*. CPA treatments, particularly CPA-L and CPA-M, partially restored the balance by reducing the overabundance of these families.

At the genus level (Figure 9L), *Bacteroides*, *Lachnospiraceae UCG-014*, and unclassified genera were the most abundant. In the model group, *Bacteroides* decreased significantly, while *Lachnospiraceae UCG-014* increased in the CPA-L and CPA-M groups. Additionally, *Romboutsia* showed a significant increase in the CPA-H group.

At the species level (Figure 9N), *Bacteroides sartorii*, *Ruminococcus flavefaciens*, and unclassified species were the most abundant taxa. CPA-L and CPA-M groups exhibited a noticeable increase in unclassified species within *Lachnospiraceae*, while the model group showed a reduction in *B. sartorii*.

### 3.13 CPA influences the differences in microbial communities between different treatment groups

In the control group, the relative abundance of *s_human_gut* and *g_Psychrobacter* was significantly higher compared to other groups. The model group exhibited an enrichment of *s_Bacteroides_sartorii* and *g_Ruminococcus_torques_group*. The Berberine treatment group showed significant enrichment of *g_Akkermansia*. The enrichment of *s_Bacteroides_stercoris* and *s_Alistipes_finegoldii* characterized the CPA-M group (Figures 9O,Q). Further analysis of the CPA dose effect (Figures 9P,R) indicated that the CPA-L group had a higher relative abundance of *s_Bifidobacterium_animalis*. The CPA-M group showed enrichment of *s_Fusobacterium_periodonticum* and *g_Morganella*. The CPA-H group exhibited an enrichment of *g_Rikenella*, *s_Bacteroides_sartorii*, and *s_Campylobacter_hominis*.

## 4 Discussion

This study comprehensively evaluated the therapeutic effects of CPA on IBS-D, focusing on macroscopic features and the underlying mechanisms. During *in vivo* experiments, we assessed the impact of the disease on overall physiological function by observing changes in rat body weight, AWR scores, Bristol stool scale scores, and thymus and spleen indices (Zhang et al., 2024; Qian et al., 2023). *In vitro* cell experiments, we explored disease effects at the cellular level, observing changes in the morphology and density of NCM460 cells, indicating cellular dysfunction and viability reduction possibly linked to intestinal inflammatory dysregulation. Furthermore, histological analysis provided macroscopic insights into tissue structure, assessing colonic tissue integrity and inflammatory cell infiltration. The results revealed significant abnormalities in the model group rats, including decreased body weight, elevated AWR and Bristol scores, altered thymus and spleen indices, disordered colonic tissue, inflammatory cell infiltration, and abnormal NCM460 cell morphology with decreased density (Zhou et al., 2024a; Zhou et al., 2024b; Qian et al., 2023). In contrast, the CPA treatment groups showed significant improvements across these macroscopic indicators, with varying degrees of colonic tissue structure recovery and a trend toward normal NCM460 cell morphology and density, particularly pronounced in the CPA-H group. These findings strongly suggest that CPA exerts its therapeutic effects on IBS-D via multiple pathways, encompassing the regulation of overall physiological function, alleviating IBS-D-related clinical symptoms, repairing tissue structure, reducing colonic inflammation, and protecting cell function.

Building on this broad assessment, the study further investigated the association between inflammatory response mechanisms and neurotransmitter dysregulation in the IBS-D model, evaluating the therapeutic effects of berberine and CPA at different concentrations. To achieve this, ELISA and qPCR techniques were employed for quantitative analysis. ELISA was used to precisely measured concentration changes of cytokines (IL-4, IL-10, IL-13, TNF-α, IL-6) and neurotransmitters (NP-Y, 5-HT), which are critical molecules in intestinal inflammation and nerve function (Wu et al., 2022). qPCR, a highly sensitive method, revealed dynamic changes in mRNA expression levels of related cytokines, reflecting transcriptional regulation of the inflammatory response. The results showed that the model group exhibited a significant pro-inflammatory response and neurotransmitter dysregulation compared to the control, specifically with elevated pro-inflammatory cytokines (TNF-α, IL-6) and reduced anti-inflammatory cytokines (IL-4, IL-10, IL-13) and neurotransmitters (NP-Y, 5-HT). This validated the model’s effectiveness and emphasized the critical role of intestinal inflammation and neurotransmitter dysregulation in the IBS-D pathogenesis. Both Berberine and CPA treatments significantly reversed this imbalance, suggesting a regulatory role in intestinal inflammation and nerve function. Notably, *in vitro* cell experiments indicated CPA reduced mRNA expression of pro-inflammatory cytokines and increased anti-inflammatory cytokine mRNA, demonstrating its potential to promote cell viability. These findings strongly suggested that berberine and CPA possess potential anti-inflammatory and neuromodulatory effects, potentially via regulation of inflammatory cytokine expression and restoration of neurotransmitter homeostasis.

Expanding on CPA’s inflammatory regulatory effects, the study further focused on its ameliorative effect and potential mechanisms on intestinal barrier function within the same IBS-D model. Histological staining and immunofluorescence techniques were combined to achieve this. AB-PAS staining was used to evaluate changes in acidic and neutral mucus secretion in colonic tissue, and this evaluation directly reflecting the protective capacity of the intestinal barrier, which is often compromised in IBS-D (Johansson and Hansson, 2022). Concurrently, immunofluorescence (IF) quantitatively analyzed expression levels of tight junction proteins (Claudin-1, Occludin and ZO-1) in colonic tissue, these proteins being critical for maintaining intestinal barrier integrity by preventing leakage (Qian et al., 2023). Results revealed significantly reduced colonic mucus secretion and decreased tight junction protein expression in the model group, indicating impaired intestinal barrier function consistent with IBS-D pathology. *In vitro*, LPS treatment decreased tight junction protein expression in NCM460 cells. However, CPA treatment significantly increased mucus secretion and upregulated tight junction protein expression, effectively reversing the LPS-induced decrease in NCM460 cells, indicating the ability of CPA to ameliorate intestinal barrier dysfunction. These findings suggested that CPA may restore intestinal barrier function by mitigating intestinal inflammatory responses, repairing damage to intestinal epithelial cells, and potentially modulating tight junction protein expression.

For the mechanistic study of CPA in IBS-D, we applied WGCNA to pinpoint IBS-D-associated hub genes. WGCNA is a statistical method for exploring gene expression data: it can cluster genes with high biological correlation, calculate the correlation coefficients between the clustered modules and clinical traits to clarify their association, and identify candidate biomarkers or disease targets (Gao et al., 2019). Currently, WGCNA has been widely used in the screening of disease-related genes for a variety of diseases (Zar et al., 2022). In this paper, we analyzed the GSE36701 dataset from the GEO to explore genes associated with IBS-D, and subsequently screened and identified the endoplasmic reticulum (ER) stress pathway as a central pathway. ER-stress refers to the cellular stress response triggered by the accumulation of unfolded or misfolded proteins in the endoplasmic reticulum (ER), an essential organelle responsible for protein synthesis, folding, and transport within cells. When ER function is compromised or the cell is in a pathological state, the accumulation of these proteins induces ER stress. The Unfolded Protein Response (UPR) is a series of signaling pathways activated to alleviate this stress and restore cellular homeostasis. The UPR consists of three main signaling pathways: IRE1 (Inositol-Requiring Enzyme 1), PERK (Protein Kinase RNA-like Endoplasmic Reticulum Kinase), and ATF6 (Activating Transcription Factor 6), which work together to promote protein folding, reduce protein synthesis, and enhance protein degradation. However, when ER stress is too severe or prolonged, and the UPR fails to resolve the issue, the cell initiates autophagy (Chen et al., 2023; Marciniak et al., 2022; Xu et al., 2005; Marciniak and Ron, 2006). Autophagy is an intracellular degradation and recycling process in which damaged cellular components are engulfed by autophagosomes, which then fuse with lysosomes to degrade their contents, thereby maintaining cellular homeostasis. During autophagy, key proteins such as Beclin-1, p62, Atg5, and LC3B play critical roles. Beclin-1 is an initiator of autophagy, involved in autophagosome formation; p62 acts as an autophagy receptor, recognizing and transporting target proteins to the autophagosome; Atg5 is an autophagy-related protein crucial for autophagosome expansion; and LC3B is a marker protein on the autophagosome membrane that mediates the fusion of the autophagosome with the lysosome (Marciniak et al., 2022; Marciniak and Ron, 2006; Mizushima and Levine, 2020; Liu et al., 2023). ER stress-induced autophagy clears misfolded proteins and damages ER by activating these signaling pathways, thereby supporting cell survival (Mizushima and Levine, 2020; Miller and Thorburn, 2021; Naama et al., 2023).

The gut microbiota, the most extensive micro-ecological system in the human body, is composed of trillions of bacteria, fungi, viruses, and other microorganisms, exhibiting complexity and diversity. They perform essential roles within the host, including facilitating the digestion and absorption of nutrients, synthesizing vital metabolites such as vitamins and short-chain fatty acids, regulating the development and maturation of the immune system, and defending against the invasion of pathogenic microorganisms (Takiishi et al., 2017). Recent studies have found that gut microbiota dysbiosis is closely associated with developing various diseases, such as inflammatory bowel disease, obesity, diabetes, and neurodegenerative diseases (He et al., 2024; Shaikh et al., 2023). This highlights the significant theoretical and clinical value of analyzing the structure and function of the gut microbiota and unraveling the mechanisms of their interaction with host health for maintaining overall health and preventing related diseases (Shaikh et al., 2023).

The study also concurrently investigated the regulatory effects of berberine and CPA on endoplasmic reticulum (ER) stress and autophagy, exploring their potential to alleviate related diseases (Johansson and Hansson, 2022; Naama et al., 2023; Grey et al., 2022). This study combined *in vitro* cell models and *in vivo* animal models using Western blotting and IHC. Western blotting, a particular and semi-quantitative technique, detected changes in key proteins within ER stress and autophagy pathways. IHC enabled *in situ* detection of protein expression, revealing dynamic and spatial changes in tissue environments. ER stress, a cellular stress response, and autophagy, a process for maintaining cellular stability, are often dysregulated in inflammatory bowel diseases like IBS-D, leading to cell damage and exacerbated inflammation (Sprenkle et al., 2017; Yorimitsu and Klionsky, 2007; Zhang and Kaufman, 2008; Tabas and Ron, 2011). The interplay between these pathways is complex, with ER stress activating autophagy to remove damaged components. *In vitro* experiments showed that ER stress activation initially upregulated the ER chaperone protein Bip before it progressed to the activation of the ATF4 pathway, which in turn upregulated CHOP and p-eIF2α (Chen et al., 2023; Marciniak and Ron, 2006). Concurrently, ER stress activation was associated with increased autophagy, marked by increased expression of p62, Beclin-1, Atg5, and LC3B and an increased LC3II/LC3I ratio (Yorimitsu and Klionsky, 2007; Bettigole and Glimcher, 2015; T et al., 2008). Berberine and CPA effectively inhibited the activation of ER stress and autophagy in model cells, with Western blotting showing reduced expression of key proteins. IHC results confirmed that CPA significantly inhibited autophagy-related protein expression in the colonic tissues of model animals. These findings, consistent both *in vitro* and *in vivo*, suggested that CPA may exert its therapeutic effects by regulating key proteins in the ER stress and autophagy pathways, offering new strategies for treating diseases associated with dysregulated ER stress and autophagy, particularly inflammatory intestinal diseases like IBS-D. This study indicated CPA’s potential to improve IBS-D pathology by modulating the ER stress-induced autophagy pathway.

Having established a broad therapeutic role for CPA, the study further investigated the impact of model treatment and subsequent interventions with berberine and varying CPA concentrations on gut microbiota composition and diversity. 16S rRNA gene amplicon sequencing of the V3-V4 region, combined with rigorous bioinformatics analysis, was used for high-resolution profiling of microbial communities. This investigation aimed to uncover the role of gut microbiota in the development of IBS-D and its potential association with inflammatory responses, given that IBS-D patients often exhibit reduced diversity and dysbiosis of specific species (Wu et al., 2022; Takiishi et al., 2017; He et al., 2024; Wei et al., 2024; Simpson et al., 2020). Amplicon Sequence Variants (ASVs) enabled precise analysis of CPA’s effects on particular species. The model treatment significantly disrupted gut microbial community structure, evidenced by reduced alpha diversity, an increased *Firmicutes* to *Bacteroidota* ratio, and an enrichment of the Ruminococcus_torques_group, all common in IBS-D. Additionally, the model group showed decreased levels of *Bacteroides* and increased levels of *Lachnospiraceae*. In contrast, Berberine and CPA treatments, especially CPA-M, showed restorative effects on the disrupted microbiome. Berberine enriched *Akkermansia*. The CPA-M group exhibited a partial restoration of the *Bacteroidota* phylum, a balanced Shannon diversity index, and an enrichment of *s_Bacteroides_stercoris* and *s_Alistipes_finegoldii*, along with a partial reversal of increased *Lachnospiraceae* and *Ruminococcaceae*. The CPA-L group exhibited more abundant *Bifidobacterium animali*s (Zhou et al., 2024a). However, higher concentrations of CPA-H was associated with increased *Fusobacterium periodonticum*, *Morganella*, *Rikenella*, and *Campylobacter hominis*. These alterations in alpha and beta diversity indicated significant impacts of the treatments on gut microbial structure. These findings are consistent with observations in IBS-D patients. This study demonstrated that the model induces substantial alterations in gut microbiota similar to IBS-D. Berberine and CPA, particularly CPA-M, can potentially reverse these shifts via their effects on inflammation. This suggested the therapeutic potential of manipulating the microbiome in IBS-D but also highlighted the need to optimize CPA dosage, given the increased potential for pathogenic bacteria at high concentrations. This study demonstrated the role of gut microbial dysbiosis in the pathogenesis of IBS-D and the potential of CPA in regulating microbial structure and function, providing a new avenue for developing microbiome-based therapies for this condition. An intriguing observation was the dose-dependent enrichment of opportunistic pathogens, including *Fusobacterium* periodonticum and *Campylobacter* hominis, in the CPA-H group. Both have been associated with intestinal inflammation. Notably, *Fusobacterium* species have been recognized as “bridging organisms” that link oral and gut microbial communities, promoting dysbiosis and exacerbating intestinal inflammation through epithelial barrier disruption and immune modulation (Li et al., 2025). Similarly, human-hosted *Campylobacter* species, including C. concisus and other oral-derived species, have been implicated in the pathogenesis of inflammatory bowel disease (Liu et al., 2018). This finding suggests that excessive CPA may inadvertently create a dysbiotic microenvironment. One possible mechanism is that certain pathobionts possess specific carbohydrate-active enzymes that enable them to overgrow when inulin-type fructans are abundantly available (Arifuzzaman et al., 2022). Importantly, this observation does not negate CPA’s therapeutic value but underscores the need for dose optimization: the medium dose (CPA-M) achieved a better balance between efficacy and microbial safety, whereas high-dose CPA should be used cautiously. Future studies should monitor these pathogenic shifts and explore baseline microbiome-guided dosing strategies. Beyond its regulatory effects on gut microbiota, we further compared the therapeutic profile of CPA with the positive control berberine. Compared to the positive control berberine, CPA exhibited several potential advantages. First, CPA’s prebiotic nature allows targeted modulation of gut microbiota, whereas berberine has broad antimicrobial effects that may disrupt commensals (Gasmi et al., 2024). Second, CPA demonstrated a dose-dependent efficacy with a favorable safety window, whereas berberine at high doses can cause constipation (Cui et al., 2024). Furthermore, CPA simultaneously alleviated ER stress and autophagy, addressing epithelial homeostasis directly (Zhou et al., 2024b; Fernández-Lainez et al., 2023). Given their distinct mechanisms—CPA primarily targeting the microbiota–ER stress axis and berberine exerting anti-inflammatory and antimicrobial actions—a combination therapy might produce synergistic effects. For instance, CPA could restore microbial diversity and barrier function, while berberine rapidly suppresses pathogenic bacteria and inflammation (Jael Teresa de Jesús et al., 2025). Future studies should explore combination regimens to determine whether lower doses of each agent can achieve superior outcomes with fewer adverse effects (Wade et al., 2024).

Finally, studies indicated CPA’s therapeutic effects are dose-dependent, particularly on parameters related to gut microbiota, ER stress, and autophagy, suggesting its efficacy may involve modulating these pathways. This reinforces the need for personalized treatment approaches tailored to patients’ gut microbiota and ER stress levels. Furthermore, CPA’s mechanism suggested potential efficacy in similar diseases such as inflammatory bowel disease and celiac disease, where dysregulation of ER stress and autophagy pathways is also critical (Spiller and Major, 2016). Future studies should explore CPA’s efficacy in these conditions. This research underscores CPA’s therapeutic potential and emphasizes the importance of personalized treatment, providing a foundation for future clinical applications. The findings indicated that CPA may have broad therapeutic effects by modulating ER stress and autophagy, with consideration of the influence of gut microbiota.

## 5 Conclusion

This study demonstrated that CPA is a promising treatment for IBS-D, with optimal efficacy observed at the medium dose (0.06 g/kg), targeting multiple pathophysiological mechanisms including inflammation, intestinal barrier integrity, ER stress, autophagy, and gut microbiota. However, CPA’s effects were not uniformly dose-dependent across the entire tested range: the highest dose (CPA-H) was associated with an enrichment of potentially pathogenic taxa, including *F. periodonticum*, *C. hominis*, and *Morganella*, suggesting a dose-limiting safety concern. Therefore, dose optimization is critical for the clinical translation of CPA, with the medium dose achieving the best balance between therapeutic efficacy and microbial safety. Future studies should further investigate the mechanisms underlying this non-monotonic response and establish the optimal dosing regimen for clinical application. Additionally, several limitations of this study should be acknowledged, including the relatively small sample size for histological and protein analyses (n = 3 per group for Western blotting, immunohistochemistry, and immunofluorescence), which limits the robustness of dose-dependent statistical conclusions derived from semi-quantitative methods; the exclusive use of male animals, which limits the generalizability of the findings to female subjects; the reliance on a single animal model and human cell line; the absence of fecal microbiota transplantation (FMT) experiments to establish a causal role of gut microbiota remodeling; the lack of direct gastrointestinal motility assessment (e.g., charcoal meal transit test); and the omission of specific pathway inhibitors to confirm that CPA acts necessarily through ER stress and autophagy pathways. Future research should validate these findings with larger sample sizes and use diverse models, including both male and female animals, patient-derived organoids, and genetically varied animals, to better capture IBS-D heterogeneity, and should incorporate FMT studies, motility tests, and pharmacological or genetic interventions to validate the proposed mechanisms. Further investigation is also needed into CPA’s molecular signaling pathways, particularly its effects on apoptosis, oxidative stress, mitochondrial function, and transcription factors. Clinical trials are required to optimize dosing, assess safety, and evaluate pharmacokinetics, pharmacodynamics, and potential off-target effects, as well as CPA’s impact on gut microbiota and its interaction with current IBS-D treatments. Addressing these gaps will help clarify CPA’s therapeutic potential and guide its clinical application.

## Data Availability

The original contributions presented in the study are publicly available. This data can be found here: https://www.ncbi.nlm.nih.gov/bioproject/PRJNA1529394.
