# Supplementary material for: Codonopsis pilosula inulin-type fructan CPA ameliorates diarrhea-predominant irritable bowel syndrome via regulation of ER stress-induced autophagy and modulation of gut microbiota
Source: Front Pharmacol. 2026 Sep 22;17:1715972. doi: 10.3389/fphar.2026.1715972 (PMC13638362; doi:10.3389/fphar.2026.1715972)

Original gels used in Figure 4 and Figure 8

Figure 4

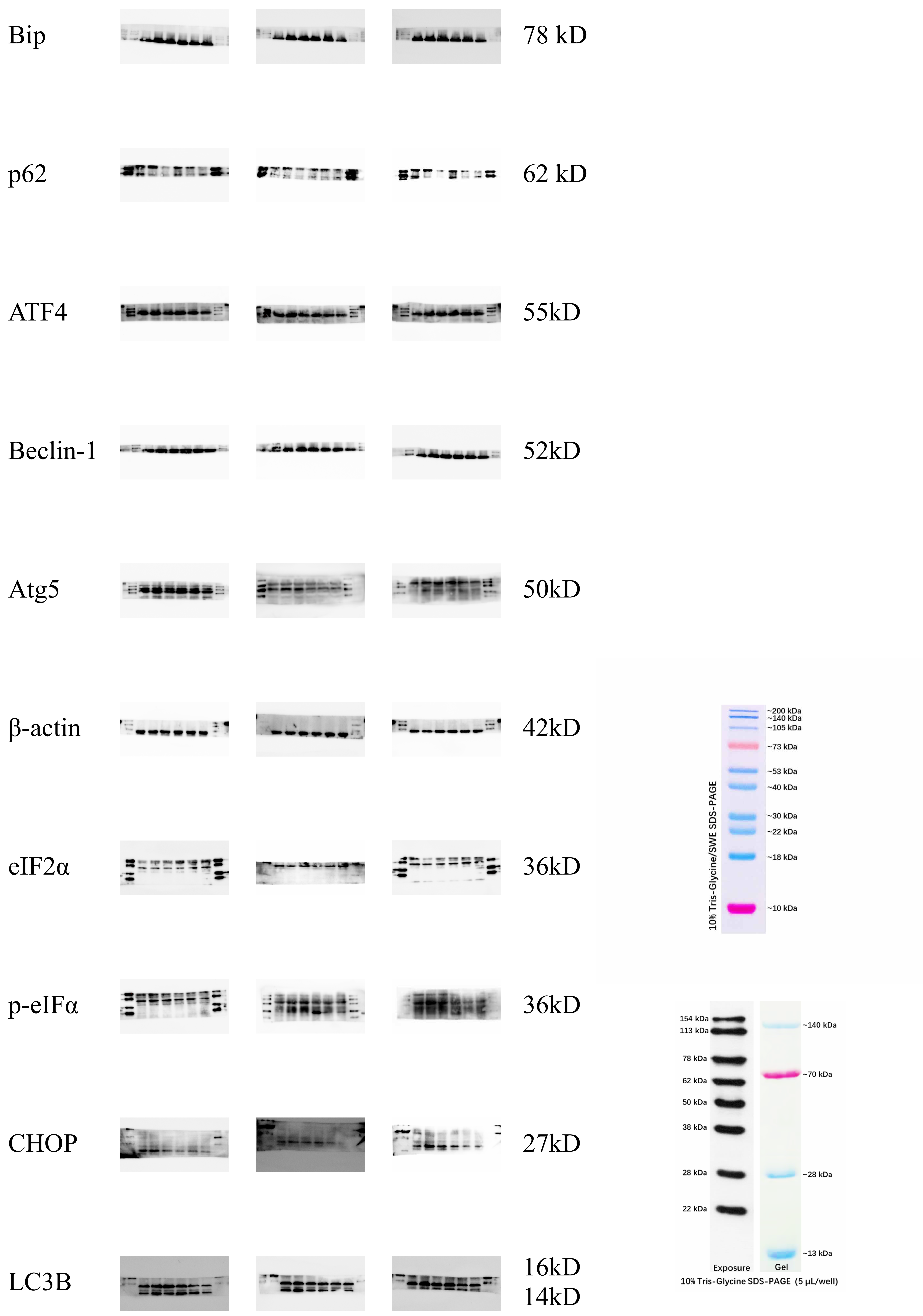

Figure 8

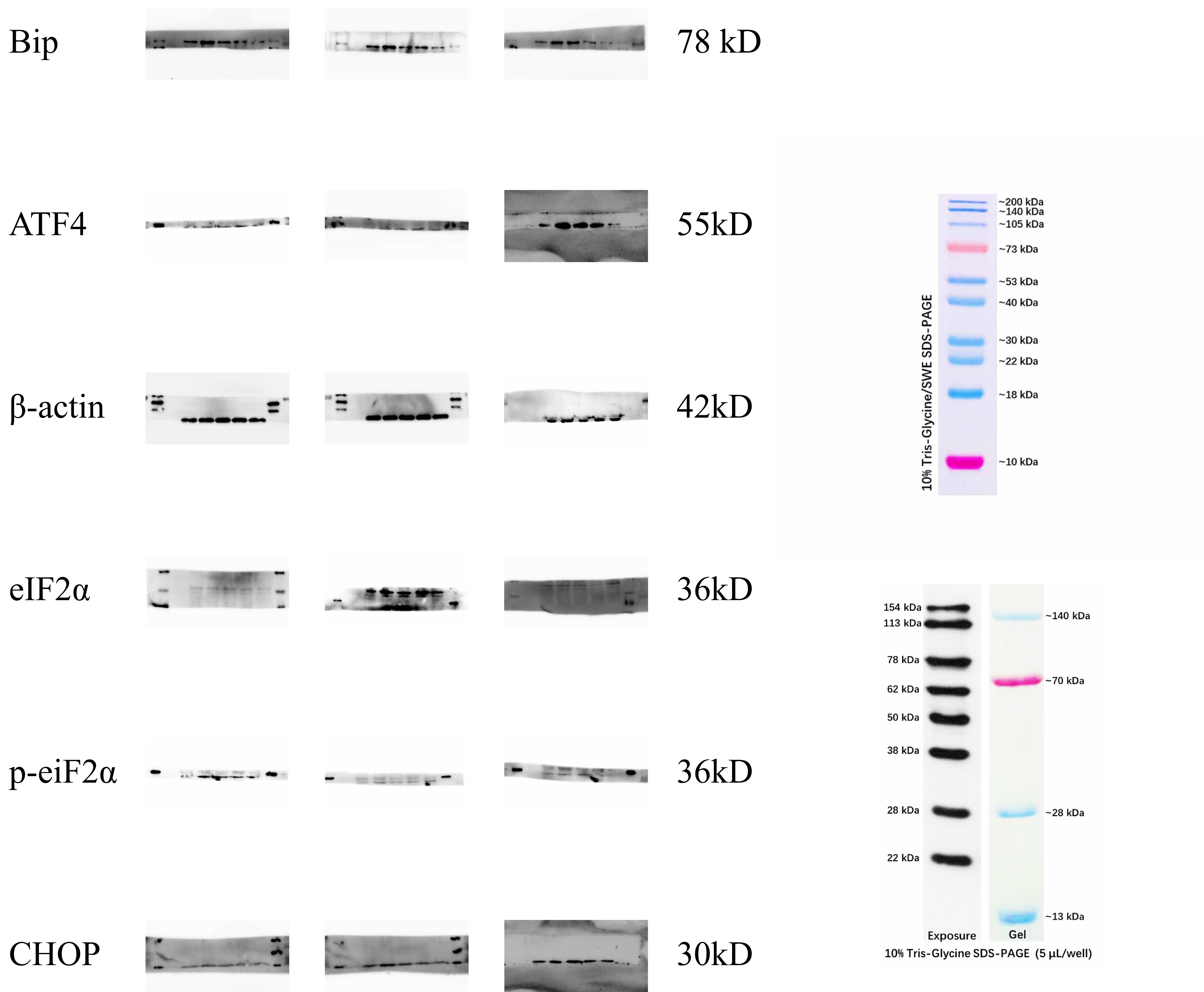

Supplement: Supplementary file 1 [file DataSheet1.pdf]
